# Factors Associated with Health-Related Quality of Life among Jordanian Patients with Diabetic Foot Ulcer

**DOI:** 10.1155/2019/4706720

**Published:** 2019-01-17

**Authors:** Ahmad Abu Alrub, Dana Hyassat, Yousef S. Khader, Radwan Bani-Mustafa, Nidal Younes, Kamel Ajlouni

**Affiliations:** ^1^The National Center for Diabetes, Endocrinology and Genetics, Amman, Jordan; ^2^Jordan University of Science and Technology (JUST), Jordan

## Abstract

**Objective:**

This study is aimed at determining factors associated with the quality of life among Jordanian diabetic patients with foot ulcers.

**Methods:**

144 consecutive patients with diabetic foot ulcers aged ≥ 18 years who were attending the diabetic foot clinic at a diabetes-specialized center were included in this study. Health-related quality of life was assessed using two self-administered questionnaires: Diabetic Foot Scale-Short Form (DFS-SF) and Short Form-8 (SF-8).

**Results:**

Patients with diabetic foot ulcer had low mean DFS-SF score and low mean scores on physical and mental component summary scales (PCS8 and MCS8). Males had significantly higher DFS-SF score indicating better health-related quality of life than females (*P* value 0.038). A patient with stressful life events had significantly lower health-related quality of life using DFS-SF scale and SF-8 summary scales. Patients with peripheral vascular disease (PVD) and patients with obesity had lower DFS-SF and PCS8 quality of life.

**Conclusion:**

Patients with diabetic foot ulcer had low quality of life. Female gender, obesity, presence of PVD, and stressful life events were the most important factors associated with lower quality of life in patients with diabetic foot ulcer.

## 1. Introduction

Diabetic foot ulcers have substantial economic burden on health care systems [1]. It is estimated that 15% of all diabetic patients will develop a foot ulcer during the course of their lifetime [2]. Diabetic foot ulcers progress to major amputation in 14% to 24% of patients [3]. The five-year mortality rate is also high, reaching 50-68% among patients who undergo major lower limb amputation [4–6]. Additionally, diabetic foot ulcers markedly increase the morbidity in patients with diabetes, leading to an increase in the number of outpatient appointments and emergency room visits as well as hospitalization days with greater risks of osteomyelitis and amputation [7–10].

Diabetic foot ulcers negatively affects patients' perceived Health-Related Quality of Life (HRQoL) due to decreased mobility and consequently the ability to perform daily activities and increasing dependence on others [11, 12]. Moreover, the perceived stress linked to wound healing or reulceration and the fear of foot amputation both increase the negative mood and lead to sleep disturbance in patients with diabetic foot ulcers [13]. Reduction of quality of life in such patients not only affects the outcome of treatment but also increases health care expenditures as a result of the frequent referring to physicians and clinical care settings [14]. Psychological comorbidity such as depression confers additional risks on diabetic patients resulting in poorer outcomes and poorer self-care. Depression in type 2 diabetes had been shown to be associated with twice the rate of first diabetic foot ulcer over 4 years of follow-up period and higher rates of amputation [15]. Moreover, depression in patients developing the first diabetic foot ulcer is associated with twofold increase of mortality over 5 years [16].

Although the impact of diabetic foot ulcers on HRQoL was studied in many countries, there is scarcity of data in Jordan on the impact of diabetes complications including diabetic foot ulcers on HRQoL. The degree by which diabetic foot ulcers impairs the quality of life is population specific. Therefore, our study was conducted to determine the impact of diabetic foot ulcers on patients' HRQoL and determine its associated factors among Jordanian patients with diabetes.

## 2. Methods

### 2.1. Study Design

A cross-sectional study was conducted among 144 patients with diabetic foot ulcers who attended the National Center for Diabetes, Endocrinology and Genetics (NCDEG) in Jordan. Patients were included in the study if their age was ≥18 years. Patients who attended the clinic twice or more during the study period of three months were interviewed during their first attendance. Pregnant or lactating women and patients with history of stroke, cancer, or mental retardation were excluded. Patients who met the inclusion criteria had been invited to participate in the study after explaining the study and its goal. All participants who agreed to participate in this study had signed the informed consent. The study was approved by the ethics committee at NCDEG.

### 2.2. Data Collection

A self-administered questionnaire was used to collect data on sociodemographic and clinical characteristics. Findings from the physical assessment were recorded in the questionnaire including presence of neuropathy symptoms, presence of peripheral vascular disease (PVD), site of the ulcer, number of ulcers, recurrence of the ulcer, duration of the ulcer, presence of previous amputation, and ulcer classification grade 1, 2, 3, 4, or 5 according to the Wagner classification [17]. Other relevant data were abstracted from the medical records including diabetes complications and comorbidity, anthropometric, and biomedical data.

### 2.3. Physical Assessment

Vascular assessment was determined by palpating dorsalis pedis and posterior tibial pulses, presence of intermittent claudication, and assessment of clinical signs and symptoms of ischemia (loss of hair, shine skin, pale skin, and skin temperature). Neurological assessment was performed for detecting the presence of neuropathic symptoms such as numbness, tingling pain, and burning sensation. Musculoskeletal assessment was performed for detecting the presence of previous amputation. Ulcer assessment included ulcer site, recurrence of the ulcer, ulcer duration, number of ulcers, and ulcer classification grade 1, 2, 3, 4, or 5 according to the Wagner classification [17].

### 2.4. Diabetic Foot Scale (DFS-SF)

The DFS is a descriptive system, which provides a comprehensive measurement of the impact of diabetic foot ulcers on patients' quality of life. The DFS consists of 29 items comprising six subscales. The six domains are leisure (enjoying life), physical health, daily activities' dependence, negative emotions, concern about wound, and wound care. After we took the permissions for use of DFS-SF from Mapi Research Trust (MRT), the questionnaire was translated into Arabic using forward-backward method. The DFS-SF subscale scores were computed based on scoring conventions published elsewhere [18]. Items were aggregated within each six subscales and then transformed to a score from 0 to 100, with higher scores indicating better quality of life for each subscale. The final version was pilot-tested among 24 patients and the necessary changes had been made. The internal consistency of subscales (Cronbach's alpha) ranged from 0.74 to 0.83. The instrument demonstrated good constructional validity when correlated with the SF-8.

### 2.5. SF-8 Health Survey

The SF-8 was developed to replicate the SF-36 version 2 with one question for each health domain. The eight domains are vitality, physical functioning, bodily pain, general health perceptions, physical role functioning, emotional role functioning, social role functioning, and mental health. Each SF-8 single-item scale and the SF-8 summary measures were scored on the same norm-based metrics as the SF-36 scales and summary measures [19]. The Arabic version which has been translated and culturally adapted in Lebanon was used [20].

### 2.6. Measurements and Definitions

Anthropometric measurements, including weight, height, and waist circumference, were measured while the subjects were wearing light clothing and no shoes. Body mass index (BMI) was expressed as the quotient between weight (kg) and height in meter squared. Patients were classified according to BMI following the recommendation of the World Health Organization as adopted by the American Diabetes Association [21]. Readings of systolic and diastolic blood pressures were taken while the subjects were seated and the arm was kept at the heart level, after at least five minutes of rest, using a standardized mercury sphygmomanometer. High blood pressure was defined as systolic blood pressure ≥ 130 mmHg or diastolic blood pressure ≥ 80 mmHg or if the patient was already on antihypertensive drugs [22]. Diabetes mellitus was diagnosed if the patient had a FPG ≥ 126 mg/dL (7.0 mmol/L) in two occasions or if the patient had a random plasma glucose ≥ 200 mg/dL (11.1 mmol/L) in the presence of classical symptoms of hyperglycemia or if he or she had HbA1c ≥ 6.5%. Moreover, diabetes was considered to be controlled if the patient had HbA1c < 7.0% according to the American Diabetes Association (ADA) 2011 guidelines [22]. Metabolic abnormalities were defined according to the American Diabetes Association 2011 [22]. Smoking was classified into three categories according to WHO guidelines 1998 [23, 24].

Retinopathy was diagnosed if it was documented by either the ophthalmologist or the treating physician in the medical records or if the patient had received laser treatment. Neuropathy was diagnosed if there was any of the following symptoms (numbness, tingling, or pain in the toes, feet, legs, hands, arms, and fingers) in patient's medical records or if the patient had done Nerve Conduction Study (NCS) which proves the presence of diabetic neuropathy or if the patient was receiving treatment for the above condition.

Stressful life event during the last year was assessed by life events, described as death, divorce, marital separation, illness, personal injury, imprisonment, dismissal from work, and retirement. Lower limb ischemia was defined as absent posterior tibial artery pulses with or without symptoms and signs of PVD or absent dorsalis pedis pulses with at least one symptom or sign indicating PVD. These symptoms and signs include intermittent claudication, edema, mottled skin, loss of hair, cold feet, and cyanotic feet. Osteomyelitis was diagnosed as physician diagnosis of osteomyelitis, which is based on radiological findings and or positive probe-to-bone test. Minor amputation refers to any amputation performed below the level of the ankle (forefoot, midfoot, or hindfoot). Major amputation refers to any amputation performed above the level of the ankle (below the knee or above the knee).

### 2.7. Statistical Analysis

Data was analyzed using the statistical program for social sciences (SPSS) version 16. Descriptive statistics used the means and standard deviations (SD) for continuous variables and used the frequency distribution for categorical variables. One-way Analysis Of Variance (ANOVA) was used to analyze the differences among group means. Multivariate Analysis of Variance was used to examine the net effect for each of the independent variable on quality of life scales and subscales. A *P* value of ≤0.05 is considered significant.

## 3. Results

### 3.1. Participants' Characteristics

A total of 144 participants aged between 24 and 90 years with a mean age (SD) of 56.8 (11.0) were included in the study. The sociodemographic, anthropometric, and clinical characteristics of the study population are presented in Tables 1 and 2.

### 3.2. Quality of Life and Subscales

The overall average score of DFS-SF was 42.1 (17.0).  Table 3 shows the mean (SD) scores of the six subscales of DFS-SF. The mean scores were 36.7 (20.1) for leisure/enjoying life, 44.2 (22.6) for physical health, 48.2 (25.7) for dependency/daily life, 43.5 (24.6) for negative emotions, 32.7 (24.2) for worried about ulcer, 46.1 (27.8) for bothered by ulcer care, 39.3 (9.9) for physical component summary-8, and 41.9 (11.1) for mental component summary-8. The summary scores showed a lower physical component summary score than mental component summary score.  Table 4 shows the mean scores of DFS-SF, PCS8, and MCS8 according to sociodemographic, clinical, and diabetic foot characteristics. Male gender, >high school level of education, no stressful events in the last year, not having PVD, absence of soft issue infection, lower Wagner classification grade, and normal body weight were significantly associated with higher DFS-SF scores, indicating better quality of life.

### 3.3. Factors Associated with the Quality of Life of Patients with Diabetic Foot Ulcer

Table 5 shows the multivariate analysis of factors associated with the quality of life scales. In the multivariate analysis, the only factors that remained significantly associated with the quality of life were gender, stressful events, PVD, and BMI. Males had significantly higher DFS-SF score indicating better health-related quality of life than females (*P* value 0.038). Patient with stressful life events had significantly lower health-related quality of life using DFS-SF scale and SF-8 summary scales. Patients with PVD and patients with obesity had lower DFS-SF and PCS8 quality of life.

Table 6 shows the multivariate analysis of factors associated with the quality of life subscales. Females scored significantly lower than males on the physical health and negative emotions DSF-SF subscales than men. Patients who had an educational level of more than high school were more worried about ulcer. Those with family income more than 500 JDs scored higher on physical health subscale. Scores in the most of DFS-SF subscales were lower in patients who had stressful life events in the last year. Patients who did not have ischemic foot ulcer had a better health-related quality of life on dependency/daily life and worried about ulcer subscales. Presence of retinopathy was associated with poor quality of life on leisure/enjoying life as well as dependency subscales. Patients with obesity scored significantly lower on bothered by ulcer care subscale.

## 4. Discussion

In this study, diabetic patients with foot ulcers had low DFS-SF, PCS8, and MCS8 scores. Diabetic foot ulcers have been shown to have a high impact on the quality of life. Ashford's study reported that families of diabetic foot ulcers patients were unable to do certain procedures, which led to family-related problems. Such problems included wound dressing, moderate mobility reduction shopping, and taking a shower and had a negative impact on patients' quality of life [25]. Goodridge et al. showed that patients with diabetic foot ulcers had a poorer physical quality of life than patients with unhealed ulcers [26]. Recent US and UK studies showed that diabetic foot ulcers adversely affect the quality of life of patients [27, 28].

Our data showed that females had significantly lower health-related quality of life than males. Women are likely to be more concerned about their health conditions and their impact on family environment than men, particularly among housewives. In agreement with our finding, most previous studies had shown that males had better health than females. Lebanese women had a lower quality of life than Lebanese men [20]. Canadian men had markedly higher scores than women in all SF-36 Health Survey domains [29]. Similarly, US men fared better than women in all SF-36 domains [30]. Except for the general health domain, British male scores were also higher than females [31]. Other studies also showed that women had a poorer quality of life [32].

PVD and diabetes often entail neuropathy, foot ulcer, increased risk of developing gangrene, ischemia, and amputation to lower extremities. Impaired lower extremity functioning is considered an important predictor of future disability and may lead to poorer quality of life [33–35]. In agreement with previous studies, patients with PVD had lower quality of life than patients without PVD; Dolan et al. found that subjects with PAD and diabetes have poorer lower extremity function than those with PAD alone [33]; Siersma et al. also reported that factors such as limb threatening ischemia, inability to stand or walk independently, and ulcer size were the most important contributors to health-related quality of life [34]. In addition, Lloyd et al. also proved that PVD in diabetic patients was significantly associated with lower physical and social functioning scale scores [35].

Our data also showed that obese diabetic patients with foot ulcers had significantly lower quality of life than nonobese diabetic patients with foot ulcers. Consistent with our result, Redekop et al.'s study also showed that obesity was related to poorer quality of life in T2DM patients [36].

Our study showed that patients with stressful life events scored lower than those without stressful life events on health-related quality of life. Stressful life events, linked to wound healing, will eventually mark an increase in the negative mood and result in improper sleep patterns [37]. Recently, many studies have illustrated the mechanism of stress in slowing the healing rate of acute and chronic ulcers, which leads to long-term ulcer care and this creates further burden, pressure, and low quality of life.

## 5. Conclusion

Patients with diabetic foot ulcer had low quality of life. Female gender, obesity, presence of PVD, and stressful life events were the most important factors associated with lower quality of life in patients with diabetic foot ulcer. Further studies are needed to assess all variables that may impact the quality of life in patients with diabetes in general and diabetic foot ulcer in particular.

## Figures and Tables

**Table 1:** Sociodemographic and clinical characteristics of the study participants (*N* = 144).

Variable	No. (%)
Age (year)	
<50	28 (19.4)
50-60	64 (44.4)
>60	52 (36.1)
Gender	
Female	42 (29.2)
Male	102 (70.8)
Marital status	
Married	120 (83.3)
Not married	24 (16.7)
Educational status	
≤high school	82 (56.9)
>high school	62 (43.1)
Health insurance	
Insured	113 (78.5)
Uninsured	31 (21.5)
Occupational status	
Employed	36 (25.0)
Unemployed	108 (75.0)
Total family monthly income (JD)	
≤500	84 (58.3)
>500	60 (41.7)
Smoking	
Nonsmoker	110 (76.4)
Smoker	34 (23.6)
Stressful events in the last year	
Yes	91 (63.2)
No	53 (36.8)
Type of diabetes mellitus	
Type 1 DM	11 (7.6)
Type 2 DM	133 (92.4)
Duration of diabetes mellitus (years)	
≤10	43 (29.9)
>10	101 (70.1)
Type of treatment	
Insulin therapy	16 (11.1)
Oral	19 (13.2)
Insulin and oral	109 (75.7)
Control of diabetes	
Controlled	23 (16.0)
Uncontrolled	121 (84.0)
Hypertension	
Yes	108 (75.0)
No	36 (25.0)
Dyslipidemia	
Yes	112 (77.8)
No	32 (22.2)
Peripheral neuropathy	
Yes	142 (98.6)
No	2 (1.4)
Peripheral vascular disease	
Yes	42 (29.2)
No	102 (70.8)
Retinopathy	
Yes	80 (55.6)
No	64 (44.4)
Coronary artery disease	
Yes	43 (29.9)
No	101 (70.1)
Body mass index^∗∗∗∗^	
Obese	83 (57.6)
Nonobese	58 (40.3)

**Table 2:** Foot ulcer characteristics among diabetic patients under treatment in the National Center for Diabetes, Endocrinology and Genetics (*N* = 144).

Variable	No. (%)
Duration of foot ulcer	
<1 month	56 (38.9)
1-3 months	37 (25.7)
>3 months	51 (35.4)
Number of foot ulcers	
1 ulcer	112 (77.8)
≥2 ulcers	32 (22.2)
Offloading device	
None	37 (25.7)
Felted foam padding	47 (32.6)
Half shoe	19 (13.2)
Removable cast walker	34 (23.6)
Total contact cast	7 (4.9)
Site of foot ulcer	
Forefoot	115 (79.9)
Midfoot	16 (11.1)
Hindfoot	13 (9.0)
Wagner classification of foot ulcer	
Grade 1	42 (29.2)
Grade 2	57 (39.6)
≥Grade 3	45 (31.2)
Soft tissue infection	
Yes	71 (49.3)
No	73 (50.7)
Osteomyelitis	
Yes	52 (36.1)
No	92 (63.9)
Recurrence of ulcer	
Yes	71 (49.3)
No	73 (50.7)
Charcot foot	
Yes	19 (13.2)
No	125 (86.8)

**Table 3:** Mean scores for the Diabetic Foot Scale-Short Form and its subscales and the two summaries of Short Form-8 (physical and mental component summaries) for the quality of life of diabetic foot ulcer patients.

Variables	QoL mean (SD)	CI (95%)
Leisure/enjoying life	36.7 (20.1)	33.4–40.0
Physical health	44.2 (22.6)	40.5–47.9
Dependency/daily life	48.2 (25.7)	44.0–52.4
Negative emotions	43.5 (24.6)	39.4–47.5
Worried about ulcer	32.7 (24.2)	28.7–36.7
Bothered by ulcer care	46.1 (27.8)	41.5–50.7
DFS-SF score	42.1 (17.0)	39.3–44.9
PCS8	39.3 (9.9)	37.6–40.9
MCS8	41.9 (11.1)	40.0–43.7

**Table 4:** Mean scores of Diabetic Foot Scale-Short Form and the two component summary of Short form-8 according to sociodemographic, clinical, and diabetic foot characteristics.

Clinical variables	DFS-SF mean (SD)	PCS8 mean (SD)	MCS8 mean (SD)
Gender			
Male	44.9 (17.5)	40.4 (9.8)	42.8 (11.5)
Female	35.4 (14.0)	36.5 (9.9)	39.7 (9.9)
*P* value	0.002^∗^	0.034^∗^	0.126
Educational status			
≤high school	39.0 (15.4)	37.3 (9.5)	39.9 (10.7)
>high school	46.2 (18.3)	41.9 (10.0)	44.4 (11.2)
*P* value	0.011^∗^	0.006^∗^	0.017^∗^
Occupational status			
Employed	45.0 (18.4)	42.1 (9.1)	43.4 (11.7)
Unemployed	41.1 (16.5)	38.3 (10.1)	41.4 (10.9)
*P* value	0.242	0.049^∗^	0.351
Stressful events in the last year			
Yes	36.9 (14.5)	37.4 (9.7)	39.7 (11.0)
No	51.0 (17.6)	42.5 (9.5)	45.5 (10.4)
*P* value	0.000^∗^	0.003^∗^	0.003^∗^
Duration of foot ulcer (month)			
<1	45.8 (17.3)	42.1 (10.0)	44.0 (10.6)
1-3	39.5 (13.6)	37.4 (10.0)	40.2 (10.1)
>3	39.9 (18.5)	37.5 (9.2)	40.7 (12.1)
*P* value	0.116	0.022^∗^	0.170
PVD			
Yes	35.8 (13.2)	35.9 (8.9)	40.1 (10.7)
No	44.7 (17.8)	40.7 (10.1)	42.6 (11.2)
*P* value	0.004^∗^	0.009^∗^	0.235
Offloading device			
None	43.9 (17.9)	41.1 (10.5)	40.2 (10.8)
Felted foam padding	47.2 (18.4)	40.0 (10.1)	45.0 (11.0)
Half shoe	40.7 (13.4)	39.1 (9.8)	40.5 (10.9)
Removable cast walker	36.4 (15.0)	37.8 (9.4)	40.2 (12.0)
Total contact cast	30.3 (5.7)	32.9 (7.0)	40.9 (6.2)
*P* value	0.016^∗^	0.280	0.223
Infection			
Yes	39.1 (15.9)	38.6 (10.3)	41.7 (10.7)
No	45.1 (17.7)	40.0 (9.6)	42.0 (11.5)
*P* value	0.035^∗^	0.400	0.886
Amputation			
Yes/minor	39.6 (17.5)	39.0 (8.4)	38.8 (11.6)
No	43.3 (16.7)	39.4 (10.6)	43.3 (10.6)
*P* value	0.227	0.812	0.023^∗^
Wagner classification			
Grade 1	48.3 (18.4)	41.0 (10.2)	44.7 (10.1)
Grade 2	40.3 (15.6)	39.6 (9.9)	40.8 (10.9)
≥Grade 3	38.6 (16.2)	37.3 (9.6)	41.6 (12.0)
*P* value	0.016^∗^	0.226	0.149
Type of diabetes			
Type 1	45.7 (20.4)	40.3 (10.1)	48.5 (13.6)
Type 2	41.8 (16.8)	39.2 (10.0)	41.3 (10.8)
*P* value	0.470	0.734	0.037^∗^
BMI			
Nonobese	46.3 (17.1)	41.6 (9.7)	43.5 (11.1)
Obese	39.5 (16.4)	37.8 (9.9)	40.8 (11.2)
*P* value	0.018^∗^	0.026^∗^	0.159

^∗^ *P* value < 0.05.

**Table 5:** Multivariate analysis of factors associated with quality of life.

Variables	Quality of life		
	DFS-SF	SF-8	
		PCS8	MCS8
Gender			
Male	44.9 (17.5)	40.4 (9.8)	42.8 (11.5)
Female	35.4 (14.0)	36.5 (9.9)	39.7 (9.9)
*P* value	0.038^∗^	0.146	0.306
Stressful life events			
Yes	36.9 (14.5)	37.4 (9.7)	39.7 (11.0)
No	51.0 (17.6)	42.5 (9.5)	45.5 (10.4)
*P* value	0.000^∗^	0.013^∗^	0.006^∗^
PVD			
Yes	35.8 (13.2)	35.9 (8.9)	40.1 (10.7)
No	44.7 (17.8)	40.7 (10.1)	42.6 (11.2)
*P* value	0.004^∗^	0.016^∗^	0.147
BMI			
Nonobese	46.3 (17.1)	41.6 (9.7)	43.5 (11.1)
Obese	39.5 (16.4)	37.8 (9.9)	40.8 (11.2)
*P* value	0.024^∗^	0.036^∗^	0.695

^∗^ *P* value < 0.05.

**Table 6:** Multivariate analysis of factors associated with quality of life subscales.

Clinical variables	Leisure/enjoying life mean (SD)	Physical health mean (SD)	Dependency/daily life mean (SD)	Negative emotion mean (SD)	Worried about ulcer mean (SD)	Bothered by ulcer care mean (SD)
Gender						
Male	38.8 (20.6)	48.3 (22.1)	50.5 (26.6)	41.4 (24.5)	47.8 (28.0)	47.1 (27.6)
Female	31.4 (18.0)	34.3 (20.9)	42.6 (22.7)	28.8 (16.5)	33.0 (20.8)	43.8 (28.5)
*P* value	0.187	0.023^∗^	0.705	0.015^∗^	0.796	0.937
Educational status						
≤high school	36.8 (20.5)	39.1 (21.2)	45.1 (23.7)	39.0 (22.0)	27.5 (20.7)	45.3 (27.0)
>high school	36.5 (19.7)	50.9 (22.8)	52.3 (27.8)	49.4 (26.7)	39.5 (26.9)	47.2 (29.0)
*P* value	0.110	0.181	0.769	0.338	0.024^∗^	0.554
Family income						
≤500	36.9 (18.4)	40.1 (22.6)	46.6 (23.8)	40.6 (23.9)	30.7 (22.1)	46.1 (28.0)
>500	36.3 (22.4)	50.0 (21.4)	50.4 (28.3)	47.5 (25.1)	35.5 (26.9)	46.1 (27.7)
*P* value	0.842	0.033^∗^	0.472	0.332	0.562	0.909
Stressful events						
Yes	32.0 (19.2)	37.1 (21.5)	42.7 (24.0)	38.0 (22.2)	29.5 (21.8)	41.4 (27.2)
No	44.7 (19.1)	56.3 (19.2)	57.5 (26.1)	52.8 (25.9)	38.2 (27.2)	54.1 (27.2)
*P* value	0.000^∗^	0.000^∗^	0.001^∗^	0.009^∗^	0.261	0.008^∗^
PVD						
Yes	33.8 (17.1)	40.6 (18.0)	37.1 (21.3)	38.2 (22.8)	24.7 (21.4)	38.4 (26.3)
No	37.8 (21.2)	45.7 (24.1)	52.7 (26.1)	45.6 (25.1)	36.0 (24.6)	49.3 (27.9)
*P* value	0.460	0.573	0.005^∗^	0.088	0.018^∗^	0.118
Retinopathy						
Yes	34.1 (17.7)	43.4 (21.1)	42.6 (24.3)	42.2 (24.7)	32.9 (24.2)	44.7 (26.5)
No	39.9 (22.5)	45.2 (24.5)	55.2 (25.9)	45.1 (24.6)	32.4 (24.5)	47.9 (29.4)
*P* value	0.031^∗^	0.327	0.007^∗^	0.605	0.323	0.886
BMI						
Nonobese	39.7 (17.7)	47.8 (23.7)	52.5 (27.5)	47.4 (23.7)	35.9 (24.1)	54.0 (27.6)
Obese	34.4 (21.5)	41.8 (22.0)	46.0 (24.2)	40.7 (24.7)	30.9 (24.5)	41.5 (26.9)
*P* value	0.401	0.346	0.652	0.275	0.421	0.022^∗^

^∗^ *P* value < 0.05.

## Data Availability

The data used to support the findings of this study are available from the corresponding author upon request.
